# Monomeric and Dimeric CXCL8 Are Both Essential for *In Vivo* Neutrophil Recruitment

**DOI:** 10.1371/journal.pone.0011754

**Published:** 2010-07-26

**Authors:** Sandhya Thulasi Das, Lavanya Rajagopalan, Antonieta Guerrero-Plata, Jiqing Sai, Ann Richmond, Roberto P. Garofalo, Krishna Rajarathnam

**Affiliations:** 1 Department of Biochemistry and Molecular Biology, The University of Texas Medical Branch, Galveston, Texas, United States of America; 2 Department of Microbiology and Immunology, The University of Texas Medical Branch, Galveston, Texas, United States of America; 3 Department of Pediatrics, The University of Texas Medical Branch, Galveston, Texas, United States of America; 4 Department of Veterans Affairs and Department of Cancer Biology, School of Medicine, Vanderbilt University, Nashville, Tennessee, United States of America; University of Georgia, United States of America

## Abstract

Rapid mobilization of neutrophils from vasculature to the site of bacterial/viral infections and tissue injury is a critical step in successful resolution of inflammation. The chemokine CXCL8 plays a central role in recruiting neutrophils. A characteristic feature of CXCL8 is its ability to reversibly exist as both monomers and dimers, but whether both forms exist *in vivo*, and if so, the relevance of each form for *in vivo* function is not known. In this study, using a ‘trapped’ non-associating monomer and a non-dissociating dimer, we show that (i) wild type (WT) CXCL8 exists as both monomers and dimers, (ii) the *in vivo* recruitment profiles of the monomer, dimer, and WT are distinctly different, and (iii) the dimer is essential for initial robust recruitment and the WT is most active for sustained recruitment. Using a microfluidic device, we also observe that recruitment is not only dependent on the total amount of CXCL8 but also on the steepness of the gradient, and the gradients created by different CXCL8 variants elicit different neutrophil migratory responses. CXCL8 mediates its function by binding to CXCR2 receptor on neutrophils and glycosaminoglycans (GAGs) on endothelial cells. On the basis of our data, we propose that dynamic equilibrium between CXCL8 monomers and dimers and their differential binding to CXCR2 and GAGs mediates and regulates *in vivo* neutrophil recruitment. Our finding that both CXCL8 monomer and dimer are functional *in vivo* is novel, and indicates that the CXCL8 monomer-dimer equilibrium and neutrophil recruitment are intimately linked in health and disease.

## Introduction

Cells interacting with pathogens in the vicinity of an infection or injury produce small pro-inflammatory molecules called chemokines, which attract and coordinate the movement of specific leukocytes towards these sites [1], [2]. The recruitment process involves chemokines interacting with heparan sulfate and related glycosaminoglycans (GAG) on the endothelial cells and in the extracellular matrix to establish concentration gradients, and activating G-protein coupled receptors (GPCR) on traveling leukocytes to effect cell shape change and extravasation into the tissue [3]–[5]. The leukocytes then travel to the site of infection, destroy the pathogens, undergo apoptosis, and are phagocytosed by tissue macrophages resulting in successful resolution of inflammation [6].

Recruitment of circulating neutrophils to the site of infection is the first line in host defense. Such a response should be immediate and robust, yet controlled. Mechanisms that spatially and temporally regulate neutrophil levels must exist in order to minimize any collateral damage to healthy tissue. If this process is not properly regulated, the infiltrating neutrophils not only kill pathogens but also destroy host tissue, a hallmark of inflammatory diseases [7].

CXCL8 (also known as interleukin-8), one of the best-characterized members of the chemokine family, recruits neutrophils under such conditions as bacterial infection and tissue injury [8]. During an inflammatory response, CXCL8 navigates through different compartments from its point of production and is taken up by venular endothelial cells abluminally and transcytosed to the luminal surface, where CXCL8 is immobilized on endothelial cell-surface GAGs for presentation to circulating neutrophils [9]. CXCL8 then provides directional cues by establishing a concentration gradient, and guides neutrophils into the underlying tissue. On reaching their destination, neutrophils augment the host defense response by many mechanisms, including release of proteases and reactive oxygen species. Clearly, multiple checkpoints in trafficking neutrophils must exist, as an imbalance in the recruitment process in terms of excess or reduced recruitment will result in tissue damage and/or failed resolution of inflammation. In this study, we show that the ability of CXCL8 to exist as monomers and dimers and their differential activities act as one such checkpoint.

More than 40 chemokines have been identified in humans, and structure determination and solution characterization have shown that dimerization is a fundamental shared property by most, if not all, chemokines. Most interestingly, chemokines show novel and complex dimerization properties that set them apart from all known proteins of similar size (MW<10 kDa). However, investigating the activities of CXCL8 (or any chemokine) monomer and dimer is not trivial as the very phenomenon of monomer-dimer equilibrium prevents studying one species without interference from the other. We circumvent this bottleneck by designing a ‘trapped’ non-associating CXCL8 monomer and a trapped non-dissociating dimer, and have shown that the structures of the trapped CXCL8 monomer and dimer are indistinguishable from those of the native monomer and dimer; therefore their functions should reflect those of the native monomer and dimer [10]–[13].

In this study, we have characterized neutrophil recruitment profiles of the monomer, dimer, and wild type (WT) CXCL8 in a mouse lung model. Our *in vivo* data show that WT exists as both monomers and dimers, and that all three variants show distinctly different recruitment profiles. At the highest dose tested, the dimer shows the highest recruitment and was the most competent in quickly mobilizing large quantities of neutrophils. On the other hand, the monomer was less active and actually showed lower recruitment at higher doses, but was active over longer time periods. In contrast, recruitment by the WT was context dependent – it showed properties resembling monomer, dimer, or distinctly unique that could not be described as either solely due to either monomer or dimer. The latter case was particularly evident at later time points, where WT was most active, indicating synergy between monomers and dimers. CXCL8 mediates its function by binding to CXCR2 receptor on neutrophils and to GAGs on endothelial cells. Our *in vivo* data reveal that differences in recruitment profiles cannot be explained on the basis of receptor affinities, suggesting that differences in GAG-binding and gradient formation, in the context of the *in vivo* milieu, dictate the overall recruitment. Our *in vitro* studies using a microfluidic device do show that recruitment is not only dependent on the total amount of CXCL8 but also on the steepness of the gradient. Our observation that both CXCL8 monomer and dimer play distinctly different *in vivo* roles is novel. We propose that CXCL8 monomer-dimer equilibrium and neutrophil recruitment are intimately linked in health and disease, and dysregulation in this process such as permanently tipping the balance towards the dimeric or monomeric form could trigger a ‘runaway’ inflammatory response resulting in severe tissue injury or failed resolution of bacterial infection leading to conditions such as sepsis.

## Results

### Recruitment by CXCL8 variants

**Figure 1:**
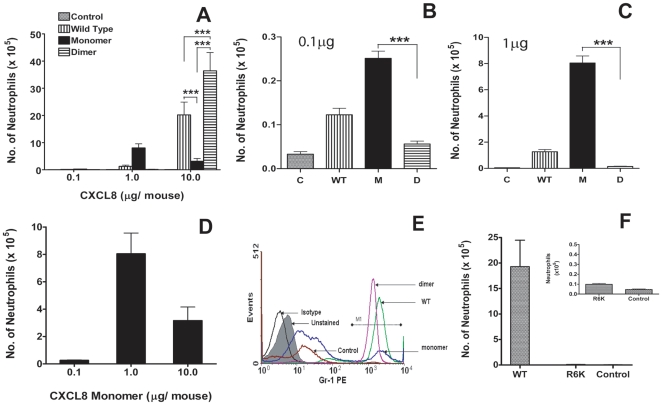
Neutrophil recruitment at 6 hours post-inoculation of various doses of CXCL8 variants. (A) Neutrophil recruitment by CXCL8 monomer, dimer, and wild type (WT) variants for 0.1, 1.0, and 10.0 µg doses. Bronchoalveolar lavage fluid (BALF) samples from mice treated with CXCL8 variants were processed as described in Methods. Each data set represents an average of 2–3 experiments using 4–6 animals/group. *P*<0.05 between monomer and dimer, monomer and WT and dimer and WT at all doses. (B–C) Neutrophil levels for 0.1 µg and 1 µg doses are shown on an expanded scale to highlight the differences in neutrophil recruitment among the different CXCL8 variants. (D) Profile of neutrophil recruitment by monomer at various doses. (E) Estimation of neutrophil levels using fluorescence-activated cell sorting (FACS). Representative data of neutrophil recruitment in lungs for the 10 µg dose are shown. BALF cells were stained with neutrophil specific Gr-1 antibody conjugated with a fluorescent dye phycoerythrin (PE). (F) Neutrophil recruitment by the inactive R6K CXCL8 mutant is negligible, and similar to the control. The inset shows levels of R6K and control recruitment on an expanded scale for better clarity. Statistical analyses were carried out using ANOVA (Graph Pad prism 4); p<0.05 between monomer and dimer, monomer and WT, and dimer and WT at all doses, and more significant if so indicated (***p<0.001).

In the current study, we have used our trapped CXCL8 monomer and dimer as surrogates to understand how the WT monomer and dimer recruit neutrophils *in vivo*. The mouse lung is a well-studied model for inflammation and infection. In order to simulate the time course of an inflammatory response, we instilled increasing doses of CXCL8 WT, trapped monomer, or trapped dimer into the lung of BALB/c mice, and measured neutrophil recruitment in the bronchoalveolar lavage fluid (BALF) at various times. We initially characterized recruitment at 6 h, as the lifetime of neutrophils is ∼6 h after which they start to undergo apoptosis and/or phagocytosed by macrophages. Our data showed that the monomer and dimer have distinct neutrophil recruitment profiles, and that their relative activities at various dosages can vary by many orders of magnitude (Figure 1A–C; Figure S1). At the lowest dose tested (0.1 µg/mouse), we observe that the trapped monomer is active, and the trapped dimer is inactive (compared to the control; p>0.05). When the dosage was increased to 1 µg/mouse, recruitment by the monomer increases 20–30 fold compared to the recruitment at 0.1 µg dose, while the dimer still remains essentially inactive. The proportion of neutrophils compared to total leukocytes also increases from ∼20 to 80% for the monomer at 1 µg, whereas it remains unchanged for the dimer (∼4%). In contrast, at the highest dose (10 µg/mouse), dimer is not only active but also shows the highest levels of neutrophil recruitment, and recruited ∼12 fold more than monomer (p<0.001). Most interestingly, monomer actually shows ∼3 fold lower activity than at the 1 µg dose (Figure 1D). The proportion of neutrophils is >95% for the dimer and ∼40% for the monomer. WT CXCL8 exhibits intermediate recruitment at all doses tested; behaving more like the monomer at lower doses and like the dimer at higher doses. The results obtained by the manual differential leukocyte count were confirmed using fluorescence activated cell sorting (FACS) (Figure 1E) and an MPO activity assay (Figure S2).

As the trapped dimer and the WT proteins are recombinantly expressed, one concern was whether endotoxins and/or other bacterial products present in our preparations contributed to neutrophil recruitment. We carried out a number of control experiments to eliminate this concern. We purified a known inactive CXCL8 mutant, R6K [14], using the same protocol used for the WT and trapped dimer, and tested for its ability to recruit neutrophils; we observe this mutant to be completely inactive and incapable of any recruitment (Figure 1F). We also measured the recruitment of a chemically synthesized trapped dimer, and observed it to be similar to that of the recombinantly expressed trapped dimer (not shown). Additionally, we measured endotoxin levels using a commercially available kit (ToxinSensor-LAL Endotoxin Assay, Genscript), and did not detect any above the background. These observations collectively indicate that all of the recruitment can be attributed to different CXCL8 variants and not to spurious contaminants.

**Figure 2:**
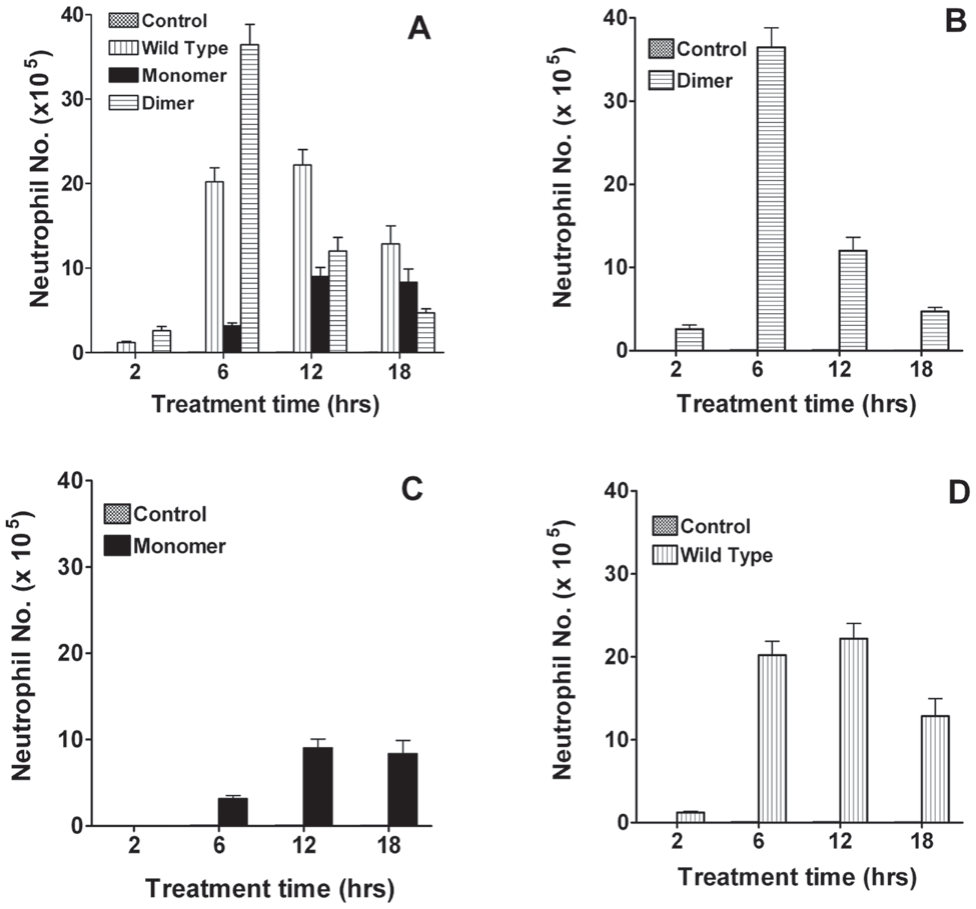
Temporal variation in neutrophil recruitment. (A) Neutrophil recruitment by CXCL8 monomer, dimer, and WT variants for the 10 µg dose at different time points. For clarity, recruitment by the individual variants is shown in panels B, C, and D. BAL samples were processed as described in Methods. Each data set represents an average of two experiments using 4–6 animals/group.

As CXCL8 variants at 10 µg dose showed a robust recruitment response, we characterized the time course of neutrophil recruitment for all variants at this dose. We observe that all three variants show distinctly different recruitment profiles (Figure 2). Whereas the recruitment by dimer is robust at 6 h, the levels drop sharply and are much lower at later time points. On the other hand, the recruitment by the monomer almost doubles at 12 h compared to 6 h, and dips slightly at 18 h. Recruitment by the WT is marginally higher at 12 h compared to 6 h, and shows lower but nevertheless substantial recruitment at the 18 h time point. Comparing recruitment at specific time points, we observe that WT is significantly more potent compared to both monomer and dimer at 12 and 18 h, and that the monomer shows relatively low activity at all time points. These data provide critical insights into temporal aspects of recruitment, and provide compelling evidence that recruitment at any given time point is context dependent, indicating that distinct recruitment profiles mediated by different CXCL8 variants will influence the outcome of how and whether inflammation will be resolved. Robust recruitment by the dimer at the 6 h time point and overall low recruitment by the monomer also indicate that recruitment is not correlated to receptor binding affinities and that binding to GAG and gradient formation most likely play a more prominent role.

### Tissue trafficking of trapped CXCL8 variants

**Figure 3:**
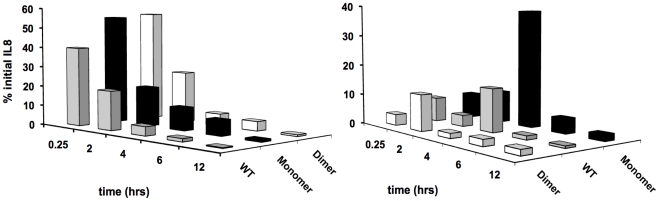
Trafficking of CXCL8 in mouse lung. Levels of CXCL8 WT (grey), monomer (black), and dimer (white) were measured in BAL (A) and serum (B) using ELISA, at different time points post-inoculation in the mouse lung. Monomeric CXCL8 shows higher serum levels (evident at the 4 h time point) than WT or trapped dimer.

CXCL8 instilled intranasally enters alveolar spaces, and then diffuses through lung tissue into the bloodstream. In order to determine whether rates of CXCL8 trafficking could influence recruitment, we determined CXCL8 levels in sera and BALF. Our data from the BALF show that all of the CXCL8 variants are rapidly transported from the alveolar spaces into the lung tissue, and CXCL8 levels are down to ∼1 to 2% of the initial amounts at the end of 12 h (Figure 3A). The data could be fitted to a simple first order kinetic equation, and indicate that the dimer and the WT are cleared from the BALF ∼2 times more rapidly than the monomer. On the other hand, we observe a more complex temporal distribution in the serum (Figure 3B). We observe the presence of all variants at the very first time point of 15 mins. However, the highest serum level is observed for the monomer at 4 h, and at this time point, dimer levels are already significantly low, and remain low for the later time points. The levels of monomer and WT rapidly fall by 6 h, and are barely detectable for the subsequent time points. If total amount of CXCL8 directly correlates with neutrophil recruitment, the chemokine levels at 4 to 6 h should be responsible for the recruitment seen at 12 h, and levels at 12 h for recruitment seen at 18 h. The higher levels seen for the monomer and the WT at 4 h crudely correlates with recruitment seen for 12 h, but there is no correlation with low CXCL8 levels at 12 h and recruitment at 18 h. These observations suggest that other factors besides absolute levels of soluble chemokine, such as the presence of a GAG-immobilized chemokine gradient, mediate overall recruitment.

### Expression of endogenous mouse cytokines and chemokines

During the process of trafficking and in the target tissue, the first wave of neutrophils trigger expression of a wide variety of molecules including expression of cytokines and chemokines. These cytokines/chemokines could recruit more neutrophils and/or recruit other leukocytes such as monocytes. It is known that monocytes are recruited as the second wave, and that these monocytes play a critical role in phagocytosing apoptotic neutrophils, and are needed for successful resolution of inflammation [15], [16]. Therefore, we measured the levels of the following mouse cytokines (IL-1α, IL-1β, IL-2, IL-3, IL-4, IL-5, IL-6, IL-9, IL-10, IL-12(p70), IL-12(p40), IL-13, IL-17, G-CSF, GM-CSF, IFN-γ) and chemokines (MCP-1, MIP-1α, MIP-1β, KC, eotaxin, and RANTES) in the BALF at different times post-inoculation with the dimer, which shows robust recruitment, and the R6K mutant, which shows no recruitment.

**Figure 4:**
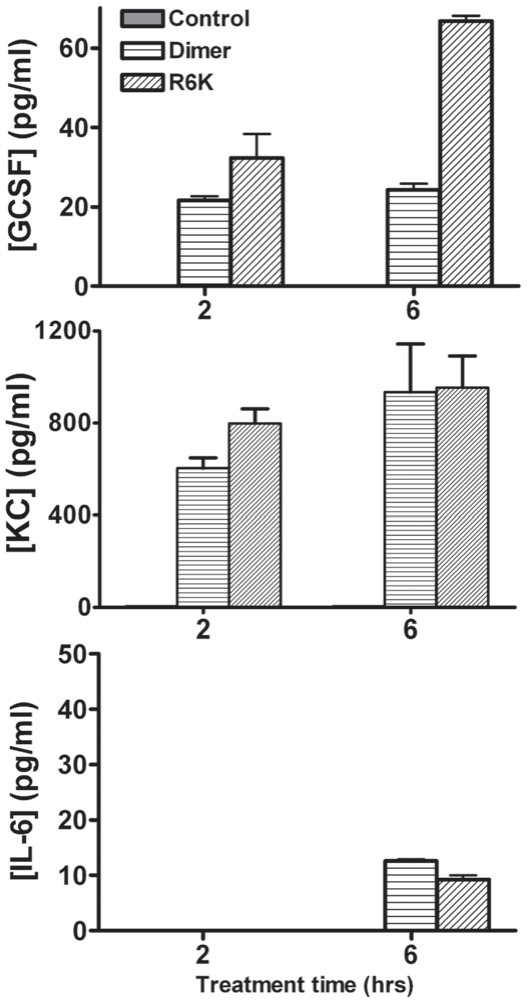
Expression of endogenous mouse chemokines and cytokines. Cell-free supernatants of BAL from mice treated with CXCL8 variants were analyzed for mouse cytokines and chemokines at different time points. Representative data of GCSF, KC and IL-6 levels from mice treated with 10 µg of trapped dimer (which shows robust recruitment) and R6K mutant (inactive, no recruitment) are shown for 2 and 6 h time points. No correlation is seen between KC levels and neutrophil recruitment profiles.

Mouse chemokines and cytokines were not elevated for 0.1 and 1 µg doses at any time point and for the 10 µg dose at 12 and 18 h time points (data not shown). However, significant expression of KC was observed for the 10 µg dose at 2 and 6 h time point for the dimer, and unexpectedly, also for the R6K mutant (Figure 4). As the R6K mutant is inactive for CXCR2 receptor activation and does not recruit neutrophils, upregulation of KC must be due to activation of a non-canonical receptor in one or more cell types in the lung. Other chemokines have been shown to activate receptors such as tyrosine kinases, but our data show such activation and subsequent signaling events play no role in neutrophil recruitment. We also observed increased levels of the cytokines G-CSF and, to a smaller extent, IL-6, for both the dimer and the R6K inactive mutant, indicating once again that the increased expression of these cytokines/chemokines play no role in neutrophil recruitment.

The observation that there is no correlation between endogenous mouse cytokine/chemokine levels and neutrophil recruitment suggest that CXCL8 continues to play a role in recruitment though the actual CXCL8 levels are barely detectable at 6 h. This could mean that CXCL8 is mostly immobilized and so could not be detected and/or that CXCL8 levels that were present at early time points has set the molecular machinery in place for subsequent overall recruitment. For instance, we observe significant levels of neutrophils in the lung tissue at 6 hrs that would eventually travel into the alveolar spaces and be detected in BALF at later time points.

Unlike monocytes, neutrophils once in the tissue are not known to traffic back into circulation and are believed to undergo apoptosis and/or phagocytosed by monocytes. The relatively higher levels of neutrophils for the monomer and WT at 12 h indicate that the rate of neutrophil trafficking from vasculature to the tissue are different for the monomer and dimer. The dimer seems to mobilize neutrophils quickly and efficiently whereas mobilization by the monomer is more persistent though at lower levels over a longer period of time.

### Inflammatory Response and Tissue Damage

**Figure 5:**
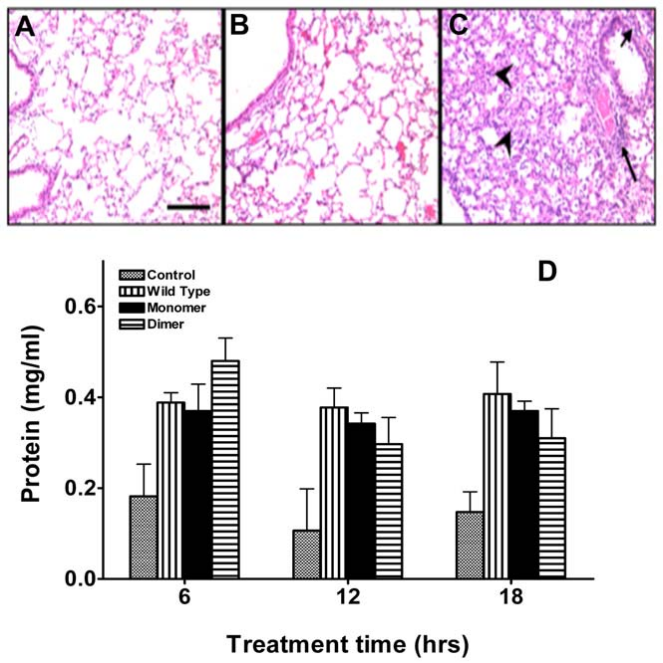
Tissue damage due to CXCL8-mediated neutrophil influx. Representative lung sections of mice treated with (A) 50 µl phosphate buffer saline (PBS), (B) 10 µg CXCL8 dimer in 50 µl PBS after 6 h, and (C) 10^7^ PFU of human metapneumovirus in 50 µl PBS after 24 h. Lungs were fixed with 10% formaldehyde in PBS, and histological sections were stained with haematoxylin and eosin (20× magnification; scale bar = 200 µm). Markers of tissue damage such as airway obliteration (arrow heads), peribronchiolar (short arrow), and perivascular (long arrow) inflammatory cell accumulation are obvious in the virus-infected lung tissue, and are completely absent in CXCL8 dimer treated lung tissue. (D) Total protein in cell free BALF from mice treated with 10 µg of CXCL8 WT, trapped monomer at various time intervals. Statistical analyses show no significant differences in protein levels among different CXCL8 variants at all time points.

To understand the consequence of increased neutrophil recruitment into the lung, we compared the histopathology of lung tissue of CXCL8 dimer-treated mice after 6 h and those infected with human metapneumovirus (hMPV), a respiratory virus that induces lung injury in infected mice after 24 h [15]. Whereas, hMPV-infected mice show evidence of damage such as airway obliteration, extensive peribronchiolar and perivascular inflammatory cell accumulation (Figure 5), CXCL8-treated mice were observed to be no different from the control.

We also measured total protein levels in BAL, as acute lung injury and tissue damage causes influx of protein into the air spaces as a consequence of increased permeability of the alveolar–capillary barrier. CXCL8-treated mice showed ∼2-fold increase in total protein for all variants at 6 h which remained essentially the same over 18 h (Figure 5D). These data indicate minimal or no neutrophil-mediated injury, and further, mice were completely healthy and showed no weight loss or other signs of stress emphasizing that these experiments mimic a proinflammatory response.

### Resolution of inflammation

**Figure 6:**
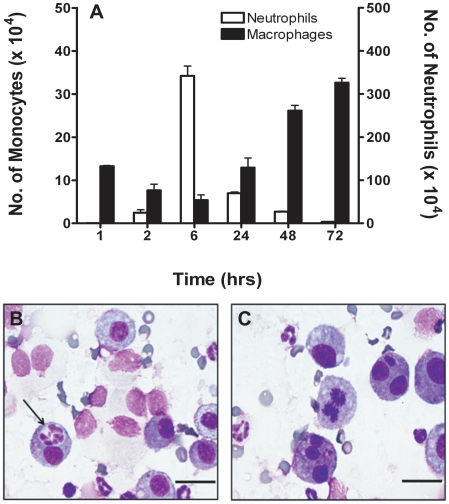
Resolution of Inflammation. (A) Monocyte and neutrophil levels in BALF of mice treated with 10 µg of dimer over a period of 72 h. Dynamics of alveolar monocytes and neutrophil levels indicate resolution of the inflammation. (B) Phagocytosis of neutrophils by monocytes (arrow) from mice treated with 10 µg dose of trapped dimer for 24 h. (C) Alveolar monocytes in various stages of mitosis are seen in BALF of mice treated with 10 µg dose of trapped dimer for 48 h. The observed cell division could explain the increase in monocytes numbers observed. Scale bar = 20 µm.

To understand how inflammation is resolved, we characterized neutrophil and monocyte levels over a period of 72 h in mice treated with 10 µg of CXCL8 dimer. We observed that neutrophil levels are maximal at 6 h and then drop dramatically. On the other hand, the alveolar monocyte numbers initially decreased up to 6 h and then continued to increase up to 72 h, and showed extensive phagocytosis of neutrophils at 24–48 h indicating resolution of inflammation (Figure 6A–B). It is well established that initial wave of neutrophils is followed by a second wave of monocytes, and that these monocytes and monocyte-derived cells undergo apoptosis and also emigrate through lymph nodes [16]. Interestingly, we also observed these monocytes/macrophages undergoing mitosis during this time period (Figure 6C). Though macrophages are differentiated cells, there is evidence that they can undergo mitosis [17], and that the overall increase in monocyte population is due to both trafficking in and out of the tissue and cell division.

### Chemotaxis using a microfluidic device

**Figure 7:**
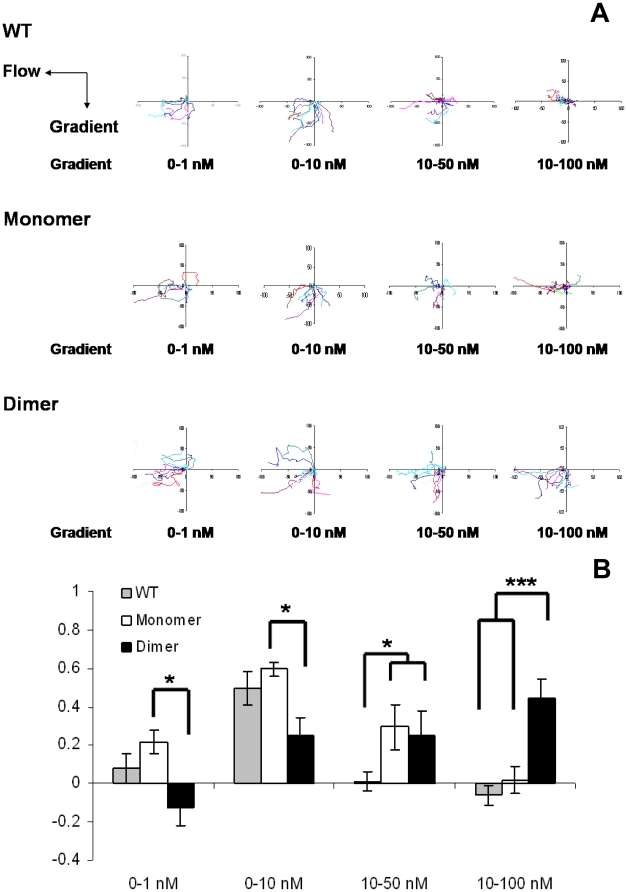
Chemotactic activity of CXCL8 variants. (A) Chemotaxis of dHL60-CXCR2 cells, in response to gradients of CXCL8 variants, was measured in the microfluidic gradient chambers. Chemotaxis for all variants was measured as a function of both varying steepness and concentration. The data are shown as the mean chemotactic index (C.I.), which is defined as the displacement of cells that move along the Y-axis (direction of gradient) divided by the total migration distance. Statistical analyses were performed with two-way ANOVA with Bonferroni posttests, *p<0.05; ***p<0.001. (B) Movement tracks of ten randomly picked cells in response to different concentration gradients of CXCL8 variants. Gradients of monomer, dimer or WT CXCL8 were delivered into the device by a constant flow (the direction shown by the arrow). The cell movements (Supplemental Videos S1 and S2) were recorded every 20 sec for 30 min and data were analyzed with Metamorph software.

To understand the mechanisms underlying how monomers and dimers mediate recruitment, we measured the chemotactic activity of our trapped monomer, dimer, and the WT for CXCR2 expressed in HL60 cells using a microfluidic chamber that allows control of both the steepness of the chemokine gradient and protein concentration [18] (Figures 7A–B; supplemental Videos S1 and S2). Monomer and WT CXCL8 are minimally active and the dimer is inactive under conditions of a shallow gradient and low chemokine concentration (0–1 nM). In contrast, both monomer and WT are maximally active under conditions of a steep gradient and low chemokine concentration (0–10 nM). At these concentrations, WT exists predominantly as a monomer, so it is not surprising that WT behaves like the monomer. Under conditions of a steep gradient and high chemokine concentration (10–100 nM), only the dimer is active while both the monomer and the WT are inactive. Lack of activity for both the monomer and the WT was unexpected. Interestingly, under conditions of intermediate gradient and high chemokine concentration (10–50 nM), dimer is less active, monomer is more active, and the WT remains inactive. The activity (or lack thereof) of the WT activity cannot be explained simply as sum of monomer and dimer activities indicating that chemotaxis is mediated by a combination of monomer/dimer ratio, total local concentration, and the steepness of the gradient. Our microfluidic assay recreates a gradient under soluble flow conditions, and any immobilization of chemokine occurs on precoated fibronectin and not on GAGs. Binding to fibronectin is not the same as GAGs, and this may also account for discrepancies between *in vivo* and *in vitro* activities of our variants. Nevertheless, we observe that our *in vitro* chemotaxis measurements of monomer and dimer correlate with some, but not all, of our *in vivo* observations. The most important observation from both studies is that higher chemokine concentrations do not translate to more robust chemotaxis. Low levels of chemotaxis and lack of recruitment for the dimer observed for shallow gradient and low concentration reflect the *in vivo* response to the 0.1 µg dose. Robust chemotaxis observed for the dimer at steep gradient and high concentration reflect recruitment observed for the 10 µg dimer at the 6 hr time point.

## Discussion

CXCL8 function *in vivo* is dictated by many factors, and most importantly, will be modulated by variations in its concentrations. Under conditions of active neutrophil recruitment, local *in vivo* CXCL8 concentrations will vary by many orders of magnitude, and so at any given time and place, it could exist as a monomer, dimer, or both. CXCL8 was the first chemokine to be discovered and characterized [19], and was initially thought to be active as a dimer as structural studies revealed CXCL8 to be a dimer [20]. Indeed, most early structural studies of various chemokines indicated that they exist as dimers and tetramers [21], [22]. CXCL8 binds its receptors with nanomolar (nM) affinity, and we had shown that a trapped CXCL8 monomer and WT CXCL8 have similar receptor binding activities [10]. It is now known that WT CXCL8 dimer dissociates at µM concentrations, and so it is not surprising that it is monomeric at nanomolar concentrations used in *in vitro* functional studies and dimeric at millimolar concentrations used in structural studies [23]. However, these studies do not rule out the possibility that dimer could bind and activate the receptor, and therefore knowledge of receptor-binding characteristics of the dimer is also essential.

To better understand the role of monomers and dimers in receptor activation, we recently characterized the functional responses of our trapped monomer, trapped dimer, and WT in mammalian cells individually expressing CXCR1 or CXCR2 [13]. We observe the dimer binds CXCR2 receptor with lower affinity and is less active for functions such as endocytosis and as active as the monomer for functions such as receptor phosphorylation and internalization. In contrast, the monomer, compared to the dimer, binds CXCR1 with higher affinity and is more active for all functional activities. Unlike human neutrophils that express both receptors, mouse neutrophils seem to express only the CXCR2 receptor. Various studies have shown that mouse does code for the CXCR1 receptor as evident from mRNA expression, but at this time, there is no evidence for expression of a functional CXCR1 receptor in mouse neutrophils [24]–[26]. Moreover, neutrophils isolated from CXCR2 knockout mouse show no chemotactic activity to both human and mouse chemokines [27], and CXCR2-specific inhibitor significantly reduces neutrophil recruitment in a number of mouse disease models [28]–[30]. These observations together suggest that CXCR2 predominantly mediates neutrophil receptor function.

Sequence and functional analysis indicate that both human and mouse express a number of neutrophil-activating chemokines. All share considerable sequence homology, have the characteristic ELR motif in the N-terminus, and show robust *in vitro* activity to both human and mouse CXCR2 expressed in mammalian cell lines. Interestingly, a mouse equivalent of CXCL8 does not exist as none of the mouse ELR-chemokines show robust activity for either human or mouse CXCR1, and it has been proposed that GCP-2/CXCL6 fulfils this role in mice [26].

CXCL8 function also involves binding to GAG on endothelial cells and the extracellular matrix, and there is evidence from *in vitro* and knockout animal model studies that GAG-binding promotes and facilitates formation of a concentration gradient, which is essential for directional neutrophil recruitment [31], [32]. Further, *in vitro* and *ex vivo* studies have shown that GAG binding and dimerization are coupled, and that the dimer binds GAGs with higher affinity [33]–[35]. *In vitro* functional measurements are carried out under controlled and steady state conditions using defined concentrations, whereas *in vivo* conditions are more complex with concentrations that could vary by many order of magnitude spatially and temporally, and so correlating *in vitro* observations to *in vivo* directional neutrophil recruitment is not trivial. Indeed, experiments using microfluidic device that allow varying CXCL8 gradients and concentrations show complex chemotaxis profiles (Figure 7).

Studying the *in vivo* roles of WT monomer and WT dimer is not feasible as the relative proportions of monomer and dimer will vary temporally and spatially, and moreover, no techniques exist to continuously monitor the levels of the two forms. In this study, we circumvent these roadblocks, by carrying out *in vivo* animal model studies using our trapped non-associating monomer and non-dissociating dimer. We observe that recruitment is context dependent, and recruitment at any given time point and dose is a snap shot but is a culmination of all events from the time point of administering CXCL8. We observe that the dimer is the most potent form for eliciting robust neutrophil recruitment, that the monomer is less potent and shows low sustained recruitment, and that the WT alone shows sustained and steady levels of recruitment, indicating a fundamental role for monomers and dimers in regulating recruitment (Figures 1 and 2). These observations are unprecedented, and could not have been predicted on the basis of *in vitro* functional assays.

**Figure 8:**
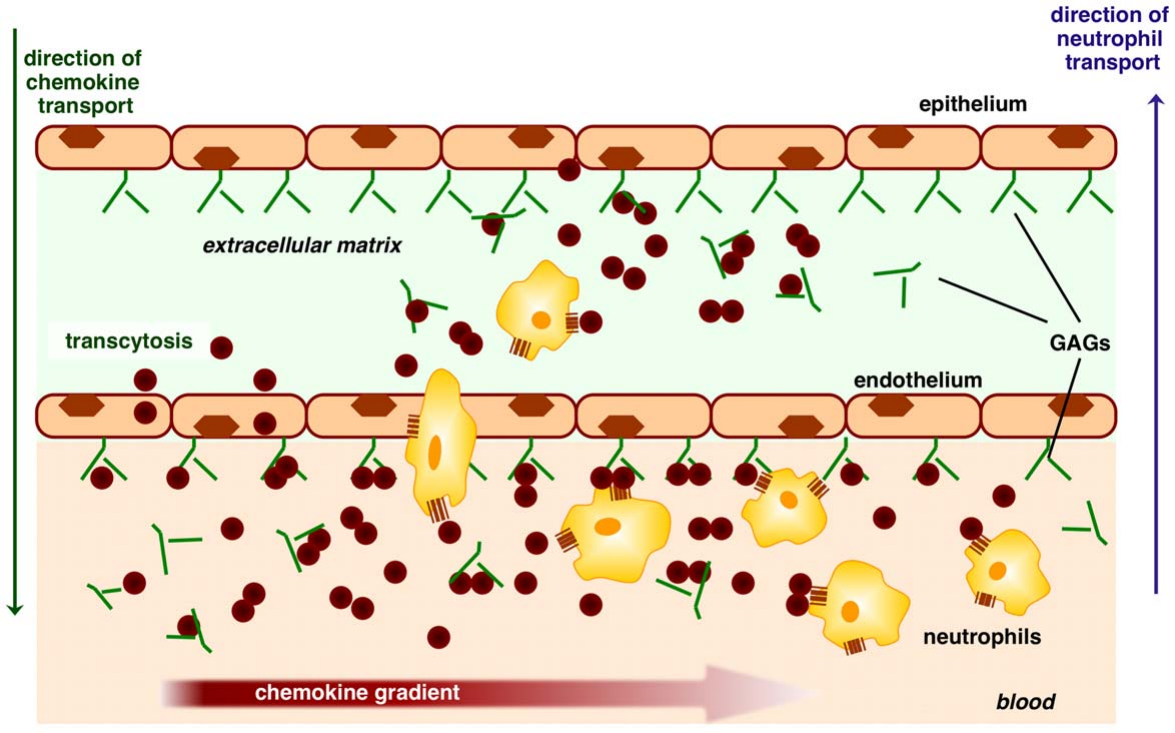
A schematic showing processes involved in neutrophil recruitment in lung tissue. CXCL8 produced at the site of insult migrates to the bloodstream where they form a concentration gradient, made up of monomers and dimers in solution, and monomers and dimers bound to GAGs. Free and GAG-bound monomers and dimers exist in equilibrium, and each species can bind and activate cognate receptors on neutrophils. Local concentrations and gradients dictate the spatial and temporal predominance of each species, which in turn modulates overall recruitment.

On the basis of our current studies, we discuss possible mechanisms of how CXCL8 recruits neutrophils (Figure 8). Our *in vitro* studies using the microfluidic device and our *in vivo* animal studies show that recruitment is not only dependent on the total amount of CXCL8, but also on the steepness of the gradient, which in turn is dependent on the continuous inter-conversion among various forms of CXCL8: free monomer and dimer and GAG-bound monomer and dimer. We observe that the ability of monomers and dimers to reach the blood stream is not limiting, and that their levels spike at different times, and also fall quickly indicating rapid clearance (Figure 3). Most importantly, we observe that there is no simple correlation between serum levels and extent of recruitment, indicating that receptor and GAG binding properties play a prominent role in regulating recruitment.

Robust recruitment by the dimer at 6 h cannot be explained on the basis of receptor binding affinity or activity alone, as dimer is equally or less active than monomer in all functional assays [13]. Lower recruitment by the monomer at the 6 h time point compared to the dimer cannot be explained on the basis of receptor affinity, as monomer is actually the high-affinity ligand [10]. Lower recruitment by the monomer at the 10 µg dose compared to the 1 µg dose also cannot be explained by receptor binding affinity (Figure 1). Therefore, interactions with GAG and gradient formation most likely play a more prominent role, with non-optimal gradients at higher concentrations leading to lower recruitment (data for the 10 µg dose at 6 h) and optimal gradients at lower concentrations leading to higher recruitment (data for the 1 µg dose for 12 h). It is also possible that the high levels of soluble monomer desensitize the receptor resulting in poor recruitment. In this case, ligand binding results in internalization of the ligand-receptor complex, proteolysis of the ligand, and eventual recycling of the receptor to the surface [13], [36]. Attenuation of recruitment has been observed in various studies where CXCL8 was systemically administered [37], [38]. It is likely that local administration of CXCL8 leads to spatially and temporally controlled gradient formation, conditions that favor directed neutrophil recruitment, whereas systemic administration results in quick uniform distribution and high soluble chemokine levels in the blood, conditions that disfavor directed neutrophil recruitment.

It is now thought that GAG-bound CXCL8 on the luminal endothelial surface forms a haptotactic gradient, which functions as a directional cue for recruiting neutrophils into the tissue [39]. It has been argued that soluble chemokine gradients are unlikely to exist, as they would be washed away with the blood flow. Nevertheless, soluble chemokine must exist due to the intrinsic equilibrium between the GAG-bound and the free forms. In principle, GAG-bound monomer and dimer, besides soluble monomer and dimer, can also bind and activate neutrophil receptors. This could be particularly relevant in the context of *in vivo* milieu due to high occupancy of the GAGs at high dimer concentrations. The only studies on GAG-bound chemokine in which CXCL8 activity was measured with and without adding soluble GAG in cell-based assays were inconclusive and contradictory with one study showing soluble GAG enhances CXCL8 binding and the other showing reduced binding [40], [41]. We propose GAG-bound CXCL8 dimer plays an important role in regulating the local CXCL8 concentration by functioning as a reservoir, and regulating soluble CXCL8 concentration for engagement with the receptor. It is essential to remember that this scenario applies only for WT dimer and not for trapped dimer. The WT dimer in the free form can dissociate to monomers and so recruit whereas the trapped dimer will not and is inactive below a threshold concentration, as reflected by its lack of activity at low doses and low activity at 12 and 18 hr time points (Figure 2).

Our data from the WT show that the ability to interchangeably exist as both monomers and dimers is essential for sustained recruitment. This is particularly evident when we correlate the recruitment at different dosages to the temporal profile of neutrophil recruitment (Figures 1 and 2). As the trapped monomer demonstrates, if WT CXCL8 did not dimerize, its recruitment though sustained would remain low. Such low levels of recruitment may not be sufficient in destroying pathogens in a timely and efficient manner that could be critical for successful resolution of inflammation. On the other hand, if WT CXCL8 exists only as a dimer, its recruitment would be robust above a concentration threshold and essentially ineffective below this threshold. These observations suggest that monomer alone cannot sustain sufficient neutrophil recruitment, and so dimerization acts as an ‘on’ switch triggering the large neutrophil influx that is essential for combating infection. However, persistent high dimer levels for extended period of time is not desirable as it could elicit a runaway inflammatory response. Data for WT CXCL8 shows sustained recruitment, indicating that the presence of both monomers and dimers regulate recruitment. We propose that the ability of CXCL8 to continuously redistribute between free- and cell-bound dimers monomers and dimers is essential for orchestrating immediate, directed, sustained, and controlled neutrophil recruitment for a healthy proinflammatory response. A schematic showing different forms of CXCL8 and their interactions for neutrophil recruitment is shown in Figure 8.

In a true disease situation, the process of resolution of inflammation is more complex due to continuous chemokine production and neutrophil recruitment that could last over a longer period. Nevertheless, our recruitment data from all of the CXCL8 variants at different doses and time points have given snapshots of how monomers and dimers and their equilibrium bring about resolution of inflammation. The level and duration of proinflammatory response needed would depend on the severity of infection. Accordingly, the amount of CXCL8 expressed could be low or high. Severe infection demands immediate response and would result in higher levels of CXCL8 expression, and is best represented by profiles seen for the 10 µg dose. Neutrophil levels observed at 6 h for the dimer are comparable to those observed in animal disease models, indicating an obligatory role for CXCL8 dimerization. Low levels of expression would essentially result only in monomers, resulting in low but sustained neutrophil recruitment and eventual resolution of inflammation. Recruitment profiles observed for the 0.1 and 1 µg doses could reflect such a scenario. On the other hand, low levels of recruitment in the course of severe infection would indicate a dysregulation in chemokine expression, and if unchecked could result in significant morbidity and mortality.

Our observation that both CXCL8 monomers and dimers can actively recruit is novel, and different from what has been observed for other chemokines. Although CXC and CC monomers are structurally similar, CXC and CC dimer structures are different as they dimerize using different regions of the protein, and oligomers show structural properties of both CXC dimers and CC dimers suggesting that the molecular mechanisms by which any given chemokine recruits leukocytes are likely to be different. For instance, CXC chemokine SDF-1α/CXCL12 that dimerizes like CXCL8 shows functional properties distinctly different from CXCL8. In contrast to our observation, a disulfide-linked CXCL12 was shown not capable of chemotaxis [42]. CXC chemokine IP-10/CXCL10 forms dimers and oligomers, and a trapped CXCL10 monomer was shown to be inactive in an *in vivo* mouse lung model [43]. Monomeric and GAG-binding-deficient mutants of CC chemokines RANTES/CCL5, MIP-1β/CCL4, and MCP-1/CCL2 are inactive in the mouse peritoneum model [44], and a trapped CC MIP-1β dimer has been shown incapable of binding its receptor [45]. These observations suggest that dimerization of CC chemokines is essential only for GAG-related function and monomers are essential only for GPCR-related function. Compelling evidence for *in vivo* dimer function also comes from a recent study that has shown CCL5 and CXCL4 form heterodimers, and design of peptides that disrupt this interaction inhibit atherosclerosis in hyperlipidemic mice [46]. We propose chemokines have evolved to exploit the property of reversibly existing as monomers, dimers, and higher order oligomers as a means to regulate a wide variety of physiological functions. This on/off switch offers the advantage of not requiring additional accessory proteins such as kinases for phosphorylation, and so can confer an advantage for fine-tuning spatial and temporal regulation that may not be otherwise possible.

In summary, our results from different dosages and recruitment levels at different time points for the monomer, dimer, and the WT have provided valuable snapshots on the mechanisms and the interrelationship between monomer-dimer equilibrium, differential binding affinities and activities for GAG and CXCR2 receptor, gradient formation, and recruitment and activation at the site of infection, and successful resolution of inflammation. In particular, we show that robust neutrophil recruitment requires CXCL8 dimerization, and that the ability of CXCL8 to reversibly exist as monomers and dimers and GAG binding-mediated gradient formation play critical roles in regulating neutrophil recruitment. Recruitment could also involve other interactions such as binding to non-canonical receptors, non-signaling chemokine receptor DARC, or proteolysis by leukocyte-released proteases [47]–[49]. DARC receptors expressed on erythrocytes and also on endothelial cells have been shown to function as sinks and so could differentially regulate recruitment [48]. However, their role in the context of monomers and dimers remain to be studied. Dysregulation in neutrophil recruitment and activation results in a wide variety of inflammatory diseases, and so considerable interest exists for designing drugs that could inhibit this process. We propose infection or injury triggers continuous chemokine production, tipping the monomer-dimer equilibrium in favor of dimer. The predominance of dimer results in persistent neutrophil infiltration, which in turn leads to acute or chronic inflammation and tissue injury. Therefore, design of small molecules that inhibit CXCL8 dimerization could function as drugs for a wide variety of neutrophil-mediated inflammatory diseases.

## Materials and Methods

### Ethics Statement

Mice were maintained in pathogen-free conditions in the animal research facility (ARC) of UTMB, in accordance with the NIH and UTMB institutional guidelines for animal care. Cages, bedding, food and water are sterilized before use. All animal work was approved by Institutional Animal Care and Use Committee (approval number – 0702005).

### Design and Synthesis of human CXCL8 variants

The trapped human CXCL8 monomer (L25NMe) was designed by substituting the dimer interface residue Leu-25 with N-Methyl Leu, which disrupts H-bonding interactions and introduces steric bulk about the two-fold symmetry point [10], [11]. The trapped human CXCL8 dimer (R26C) was designed by mutating Arg-26 at the dimer interface to Cys-26. This results in a disulfide bridge formation between two monomers in the dimer [12]. L25NMe was chemically synthesized, and the wild type (WT), trapped dimer R26C, and the inactive R6K mutant were recombinantly expressed.

### *In vivo* recruitment in a mouse lung model

Female, 8- to 10-week-old BALB/c mice purchased from Harlan (Houston, TX) were housed under specific pathogen-free conditions in the animal research facility of University of Texas Medical Branch (UTMB), in accordance with the National Institutes of Health and UTMB guidelines for animal care. Under light anesthesia, mice were inoculated intranasally with 0.1, 1 or 10 µg of CXCL8 variants in Dulbecco's phosphate-buffered saline (D-PBS) in a 50 µl volume [50]. Control mice were inoculated with the same volume of D-PBS. At various time points post-inoculation, mice were injected intraperitoneally with a mixture of ketamine and xylazine, and sacrificed by exsanguination via femoral vein puncture. The BALF was collected by flushing lungs three times with 1 ml of ice-cold DPBS. BALF from each animal were pooled and centrifuged at 4°C for 5 mins at 13,000 rpm, and the pellet was resuspended in 1 ml of fresh D-PBS buffer and used for cytospin slides and total leukocyte counts. Cytospin slides of BALF were prepared (Shandon, Thermo Electron Corporation; 5 mins at 800 rpm), fixed and stained with hematoxylin and eosin, and a differential leukocyte count was performed. For total leukocyte determination, BALF samples were diluted with Turk blood diluting fluid (Ricca chemical, TX) to lyse RBCs and then the total cells were counted using a hemocytometer.

For estimating neutrophils by FACS, cells were stained with a neutrophil-specific fluorescently labeled antibody against the murine myeloid differentiation antigen Gr-1 (RB6-8C5, PharMingen), and analyzed with a FACScan flow cytometer equipped with CellQuest software (Becton Dickinson).

For estimating neutrophils by myeloperoxidase (MPO) assay, cells from BAL fluid were resuspended in 50 mM phosphate buffer (pH 6.0) containing 0.5% hexadecyltrimethylammonium bromide (HTAB), sonicated, centrifuged, and the supernatants were tested for MPO activity using o-dianisidine (Sigma) and H_2_O_2_ as substrates. The change in absorbance at A_460_ was measured at 1 min intervals for 7–8 min. One unit of MPO activity is defined as the amount of enzyme which degrades 1 µmol of H_2_O_2_/min at 25°C.

### Estimation of CXCL8 levels using ELISA

Tissue trafficking of CXCL8 WT, trapped monomer, and trapped dimer were analyzed in the lung model. BAL, lung, and serum samples were analyzed using ELISA. CXCL8 levels in BAL, serum, and lung tissue samples were measured immediately after thawing and appropriate dilution in HBSS, using the IL-8/CXCL8 DuoSet ELISA Kit (R&D Systems, Minneapolis, MN). CXCL8 levels were normalized to initial levels (total levels in BAL, lung and serum, measured immediately after inoculation, equals 100%) for each CXCL8 variant, and plotted as a function of time.

### Mouse cytokine/chemokine analysis

The cell-free supernatants, collected and stored at −70°C as described above, were thawed to room temperature and tested for various mouse cytokines and chemokines using the Bio-Plex Mouse Cytokine 23-Plex panel (Bio-Rad Laboratories, Hercules, CA), according to the manufacturer's instructions. All of the cytokines/chemokines were assayed using BALF from at least two sets of mice each containing three to four different animals for any given dose and time point. The reported values are the average of all the values.

### Protein estimation

Total protein in BAL supernatants was estimated by the BCA assay using the BCA protein assay kit (Thermo Fisher Scientific Inc., Rockford, IL, USA) following the manufacturer's instructions.

### Chemotaxis assays using microfluidic device

Human promyelocytic HL-60 cells stably transfected with the CXCR2 plasmid were used for the chemotaxis assays. The HL-60 cells were differentiated by adding 1.25% dimethylsulfoxide (DMSO) to the cell suspension, and differentiation was evaluated by observing morphological changes evident 6–7 days after DMSO addition. The microfluidic chemotaxis device used in this study was especially designed to establish a stable and uniform chemokine concentration gradient [18]. The cells were monitored by time lapse imaging with a Hamamatsu digital camera using MetaMorph® software (Molecular Devices Corporation, Downingtown, PA) on a Zeiss Axiovert 200M inverted microscope (Carl Zeiss MicroImaging, Inc, Thornwood, NY), with images obtained at 20 sec intervals for 30 mins. The images obtained were analyzed using MetaMorph® software.

#### Statistics

All results unless specified otherwise are shown as mean and standard deviation from the mean. Differences were considered significant at p<0.05. All experiments were repeated at least three times. Statistical analyses were carried out using ANOVA (Graph Pad prism 4).

## Supporting Information

Figure S1 Estimation of neutrophil levels in the lung Broncheo-alveolar lavage fluid (BALF). BAL neutrophils (N) and macrophages (M) as seen in cytospin slides stained with hematoxylin and eosin (H&E), obtained from mice treated with PBS (control), CXCL8 wild type (WT), trapped monomer and trapped dimer. Scale Bar, 20 µm.
(1.85 MB DOC)

Figure S2 Estimation of neutrophil levels in the BALF using MPO assay. MPO is a neutrophil granule enzyme and its activity has been shown to correlate with neutrophil levels. Measured neutrophil levels are similar to those measured from differential counting (Fig. 1A). P is at least <0.01 between monomer and dimer, monomer and WT, and dimer and WT at all doses. Each data set represents an average of 2–3 experiments using 4–6 animals/group. Statistical analyses were carried out using ANOVA (Graph Pad prism 4).
(0.18 MB DOC)

Video S1 A video file showing neutrophil chemotaxis for the monomer.
(5.81 MB MOV)

Video S2 A video file showing neutrophil chemotaxis for the dimer.
(2.57 MB MOV)
