# Supplementary material for: Monomeric and Dimeric CXCL8 Are Both Essential for In Vivo Neutrophil Recruitment
Source: PLoS One. 2010 Jul 26;5(7):e11754. doi: 10.1371/journal.pone.0011754 (PMC2909905; doi:10.1371/journal.pone.0011754)
Supplement: Figure S1 — Estimation of neutrophil levels in the lung Broncheo-alveolar lavage fluid (BALF). BAL neutrophils (N) and macrophages (M) as seen in cytospin slides stained with hematoxylin and eosin (H&E), obtained from mice treated with PBS (control), CXCL8 wild type (WT), trapped monomer and trapped dimer. Scale Bar, 20 µm. (1.85 MB DOC) [file pone.0011754.s001.doc]

**Supplemental data**


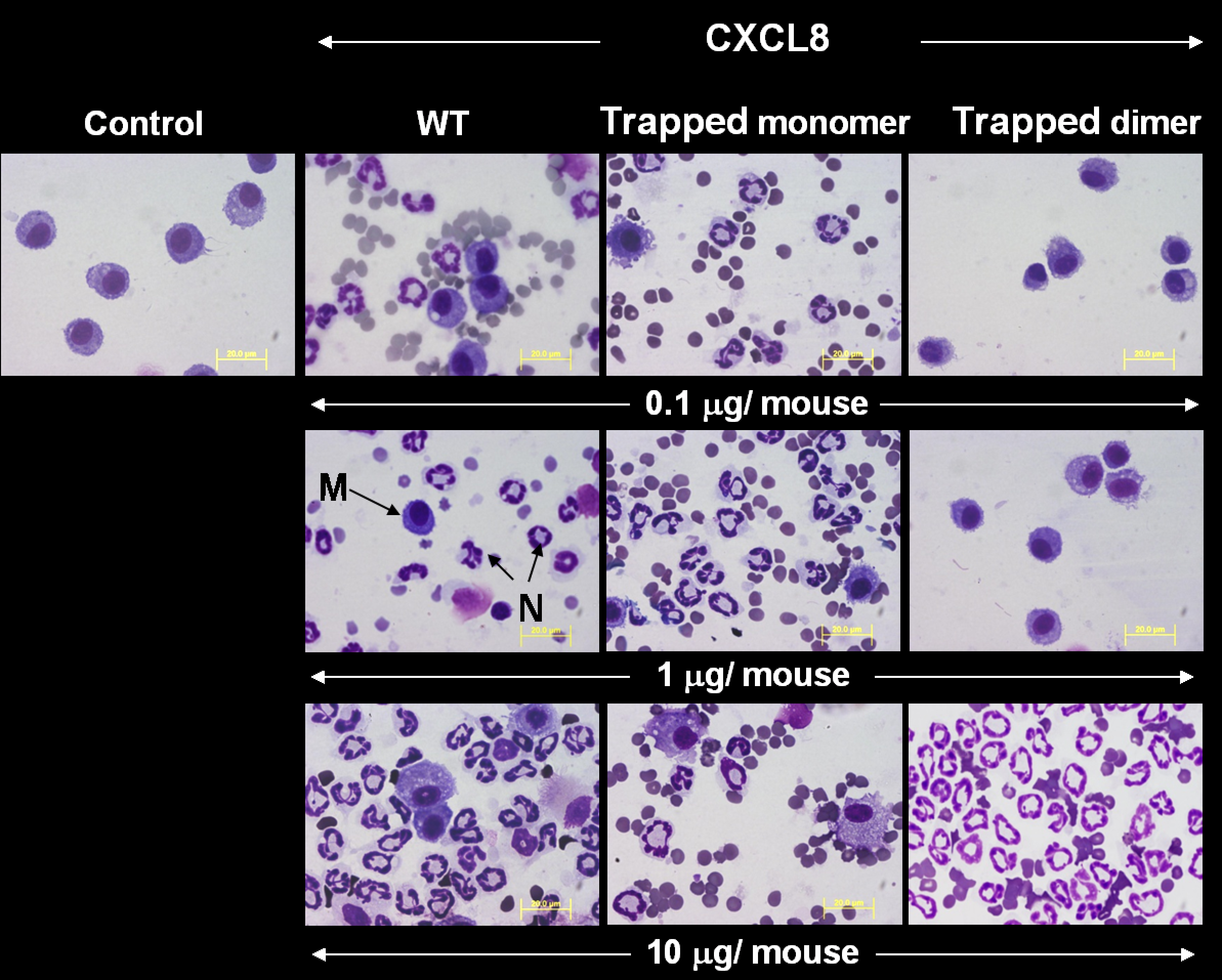


**Figure S1. Estimation of neutrophil levels in the lung Broncheo-alveolar lavage fluid (BALF).** BAL neutrophils (N) and macrophages (M) as seen in cytospin slides stained with hematoxylin and eosin (H&E), obtained from mice treated with PBS (control), CXCL8 wild type (WT), trapped monomer and trapped dimer. Scale Bar, 20 μm.
