# Supplementary material for: Monomeric and Dimeric CXCL8 Are Both Essential for In Vivo Neutrophil Recruitment
Source: PLoS One. 2010 Jul 26;5(7):e11754. doi: 10.1371/journal.pone.0011754 (PMC2909905; doi:10.1371/journal.pone.0011754)
Supplement: Figure S2 — Estimation of neutrophil levels in the BALF using MPO assay. MPO is a neutrophil granule enzyme and its activity has been shown to correlate with neutrophil levels. Measured neutrophil levels are similar to those measured from differential counting (Fig. 1A). P is at least <0.01 between monomer and dimer, monomer and WT, and dimer and WT at all doses. Each data set represents an average of 2–3 experiments using 4–6 animals/group. Statistical analyses were carried out using ANOVA (Graph Pad prism 4). (0.18 MB DOC) [file pone.0011754.s002.doc]

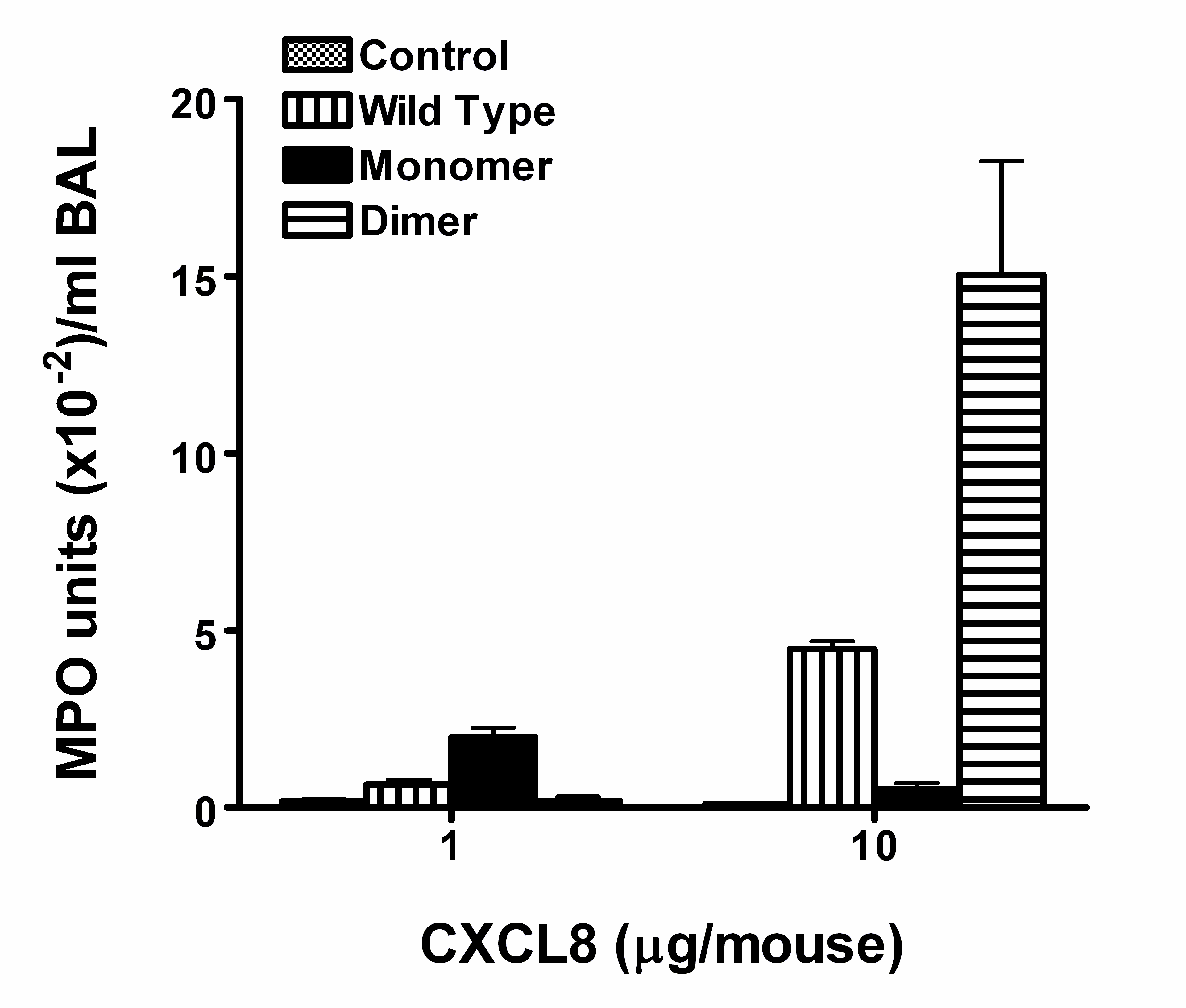


**Figure S2**. **Estimation of neutrophil levels in the BALF using MPO assay.** MPO is a neutrophil granule enzyme and its activity has been shown to correlate with neutrophil levels. Measured neutrophil levels are similar to those measured from differential counting (Fig. 1A). *P* is at least <0.01 between monomer and dimer, monomer and WT, and dimer and WT at all doses. Each data set represents an average of 2-3 experiments using 4-6 animals/group. Statistical analyses were carried out using ANOVA (Graph Pad prism 4).
